# LDL-cholesterol trajectories and statin treatment in Finnish type 2 diabetes patients: a growth mixture model

**DOI:** 10.1038/s41598-021-02077-6

**Published:** 2021-11-19

**Authors:** Laura Inglin, Piia Lavikainen, Kari Jalkanen, Tiina Laatikainen

**Affiliations:** 1 Institute of Public Health and Clinical Nutrition, University of Eastern Finland, Yliopistonranta 1, 70210 Kuopio, Finland; 2 School of Pharmacy, University of Eastern Finland, Kuopio, Finland; 3 Finnish Institute for Health and Welfare, Helsinki, Finland; 4 Joint Municipal Authority for North Karelia Health and Social Services (Siun Sote), Joensuu, Finland

**Keywords:** Health care, Risk factors, Epidemiology, Diabetes complications, Type 2 diabetes

## Abstract

We aimed to identify distinct longitudinal trends of LDL-cholesterol (LDL-C) levels and investigate these trajectories’ association with statin treatment. This retrospective cohort study used electronic health records from 8592 type 2 diabetes patients in North Karelia, Finland, comprising all primary and specialised care visits 2011‒2017. We compared LDL-C trajectory groups assessing LDL-C treatment target achievement and changes in statin treatment intensity. Using a growth mixture model, we identified four LDL-C trajectory groups. The majority (85.9%) had “moderate-stable” LDL-C levels around 2.3 mmol/L. The second-largest group (7.7%) consisted of predominantly untreated patients with alarmingly “high-stable” LDL-C levels around 3.9 mmol/L. The “decreasing” group (3.8%) was characterised by large improvements in initially very high LDL-C levels, along with the highest statin treatment intensification rates, while among patients with “increasing” LDL-C (2.5%), statin treatment declined drastically. In all the trajectory groups, women had significantly higher average LDL-C levels and received less frequent any statin treatment and high-intensity treatment than men. Overall, 41.9% of patients had no statin prescribed at the end of follow-up. Efforts to control LDL-C should be increased—especially in patients with continuously elevated levels—by initiating and intensifying statin treatment earlier and re-initiating the treatment after discontinuation if possible.

## Introduction

Suboptimal lipid profiles and particularly elevated low-density lipoprotein cholesterol (LDL-C) are strongly associated with atherosclerotic cardiovascular diseases (CVD) in individuals with type 2 diabetes (T2D)^1,2^. To prevent or at least delay complications, regular follow-up visits and good control of HbA_1c_, LDL-C, blood pressure, and other CVD risk factors are vital in diabetes management^3^. International and national guidelines have consistently identified statins as the principal lipid-lowering therapy, recommended particularly at moderate- to high-intensity^4–7^.

Real-world evidence has shown that appropriate statin use and LDL-C control may remain suboptimal in clinical practice^8–11^. In Finland, statin use has continuously increased among the general population and individuals aged over 65 years during 1995–2010^12,13^ but after that remained stable in men and even decreased in women 2010–2015, leading to an increased sex gap in statin use^13^. A recent study on trends in T2D care in the North Karelia region, Finland, between 2012 and 2017 found increases in the LDL-C treatment target achievement (< 2.5 mmol/L) from 53.4 to 59.5% and in statin prescriptions from 65.7 to 71.0%, respectively^14^.

Previous studies have investigated heterogeneity in lipid development using trajectory models and identified distinct LDL-C trajectories in different patients groups^15–17^. However, none of these studies has focused on T2D patients or examined variation in statin treatments by LDL-C trajectories.

Therefore, we aimed to identify possible gaps in current diabetes management by (1) identifying distinct LDL-C trajectory groups within T2D patients; (2) describing these groups with patient characteristics and quality of care process and outcome indicators; (3) examining the association of trajectory groups with annual statin therapy and changes in treatment intensity; and (4) capturing sex disparities in care provision.

## Methods

### Study setting

In Finland, municipalities organise public healthcare and it is primarily tax-funded. Municipalities organise services by themselves or in collaboration with other municipalities like in North Karelia, where healthcare has been managed by the Joint Municipal Authority for North Karelia Social and Health Services (Siun sote) since 2017. Public services account for about three-quarters of all services and are complemented by private healthcare^20^. Finnish residents are entitled to public healthcare for free or reasonable fees and medication reimbursement^21^. The reimbursement rates for prices of statins were 40–65%, depending on the patient’s medical condition^22^.

### Study design

We used regional electronic health records (EHRs) from Siun sote of patients in public primary and secondary healthcare services since 2011 for this retrospective cohort study. The database, called Mediatri, comprises demographic information, date of T2D diagnosis, drug prescriptions (date, Anatomical Therapeutic Chemical (ATC) codes^18^), diagnoses (ICD-10 codes^19^), and laboratory measurements. Data were retrieved for 2011–2017 from all patients living in the region diagnosed with T2D (E11) at the end of 2012 (n = 10,139). The clinical criteria for T2D diagnosis (E11) were: (i) fasting glucose tolerance ≥ 7 mmol/mol, or (ii) 2-h glucose tolerance > 11 mmol/mol, or (iii) HbA1c ≥ 48 mmol/mol^20^. Additionally, data were retrieved from two registers maintained by the Social Insurance Institution (SII) of Finland: the Finnish Prescription Register (reimbursed diabetes medication purchases for 1995–2010) and the Special Reimbursement Register (entitlements to higher medication reimbursement for diabetes medications before 2011). Person-level data linkage was performed using de-identified identification numbers. The timing of T2D diagnosis was determined based on information from the EHRs and the registers, considering the first occurrence of confirmed diagnosis.

### Variables

#### LDL-C measurements

LDL-C samples were analysed in the Eastern Finland Laboratory (ISLAB), using the photometric direct enzymatic method and standardised to the International Federation of Clinical Chemistry (IFCC) units. LDL-C values were considered valid for this study when (1) recorded after the diagnosis of T2D and (2) not recorded before the baseline LDL-C measurement, defined as the latest value during 2011–2012. We excluded patients with less than two valid LDL-C measurements during 2011–2017 or whose valid measurements were not recorded in at least two different years (n = 1547) (see Supplementary Fig. S1). Patients’ LDL-C measurements were followed until death, moving outside of the study region, or 31 Dec 2017, whichever came first. For the trajectory modelling, we used the latest LDL-C measurement of each year during 2013–2017, and calendar time in years as a metric of time (Supplementary Table S1).

### Baseline characteristics

Baseline information included age (at 31 Dec 2012), the metabolic factors BMI, HbA_1c_, and LDL-C (latest value during 2011–2012) among patients with available measurements, statin prescription (at 31 Dec 2012), concordant comorbidities diagnosed before 1 Jan 2013, identified with ICD-10 codes (Supplementary Table S2). HbA_1c_ values were measured with the turbidimetric inhibition immunoanalysis method (TINIA) in the ISLAB and standardised to IFCC units.

### Process and outcome indicators

We defined three process and three outcome indicators of quality of care based on the Finnish Current Care Guideline^23^ and diabetes care evaluation recommendations^24^. We calculated total measurement frequency and the proportions of patients having LDL-C measured annually or biennially. Furthermore, we assessed care outcomes through mean values and the achievement of the two LDL-C treatment targets < 2.5 mmol/L and < 1.8 mmol/L overall and in three different periods (2011–2012, 2013–2015, and 2016–2017). The overall LDL-C indicators were based on the mean of all valid measurements (2011–2017), whilst the remaining outcome indicators were evaluated based on the latest measurement of each period (Supplementary Table S2).

### Statin therapy

Statin and other lipid-lowering therapy prescriptions were identified from the EHRs based on ATC codes C10AA, C10AX09, C10BA, and C10BX. Patients were considered as having received statin treatment if they had an ongoing prescription on 31 Dec of each year during 2012–2017. Statin therapy was categorised into four intensity levels (no treatment, low-, moderate- and high-intensity) based on the average expected LDL-C response to a specific statin type and dose^25^ (Supplementary Table S3). The combination of any statin with ezetimibe was considered a high-intensity treatment. Other lipid-modifying agents were not taken into account.

We evaluated patients’ annual statin treatment during 2012–2017, including each year patients who were alive and residing in North Karelia by 31 Dec (Supplementary Table S4). We analysed the rate of intensification and de-intensification and time to the first change in treatment during the follow-up period, defined as a change of treatment intensity level in any of the years between 2012 and the patients’ last year of follow-up. We also compared the treatment intensity level in 2012 with the last year of follow-up by defining initiations among initial non-users, discontinuations among initial users, and the magnitude of the final treatment change, classified as “intensified” (three-staged: one, two, or three levels change), “unchanged”, and “de-intensified” (three-staged: one, two, or three levels change).

As a sensitivity analysis, we created treatment indicators that were based on statin prescriptions at any time during the year, favouring the highest treatment intensity. Treatment patterns were similar but potentially overestimated statin treatment (results not shown).

### Statistical analyses

A growth mixture model (GMM) was used to identify LDL-C trajectories, allowing within-group variation of individuals with random effects (random intercepts and slopes) to estimate variance around the growth parameters. We also fitted latent class growth models (LCGA) assuming homogenous variation within groups, but the estimated models had poorer fit with the data than the GMMs (data not shown). Both GMM and LCGA can handle values missing at random, using all available data in estimation. Linear, quadratic and cubic unconditional models were fitted from single to 5-group models with different random effect specifications. We used the following (hierarchical) criteria to select the best fitting model: (1) interpretability and clinical relevance of the models, (2) minimal group size (≥ 2.5% and n ≥ 100 per group when both sexes were analysed separately), (3) Bayesian information criterion (BIC), (4) significant Lo-Mendell-Rubin likelihood ratio test (LMR-LRT), and (5) average posterior probabilities > 0.7 in each group^26,27^. Although not used as a model selection criteria, an entropy index > 0.8 ensures that the model can clearly classify persons in a specific group with adequate between-group separation^26^.

GMMs were fitted for both sexes together as they had similar trajectories. However, due to significant sex differences regarding LDL-C levels and statin use in clinical practice, all trajectory group-specific analyses were conducted for both sexes separately.

Differences between trajectory groups, sexes, and patients included and excluded in the study sample were examined using Pearson’s Chi-Square test for categorical variables and Kruskal–Wallis test for non-normally distributed continuous variables, omitting missing values. Two-sided *P* value < 0.05 was considered statistically significant. The following softwares were used: IBM SPSS Statistics (version 26.0) to process the raw data, M*plus* (version 7)^28^ to estimate GMMs and LCGAs, and *R* (version 3.6.0)^29^ for the remaining analyses.

### Ethics statement

Use of the data was approved by the Ethics Committee of the Northern Savo Hospital District (diary number 81/2012). The study protocol was also approved by the register administrator, Siun sote. A separate permission to link data on medication purchases and special reimbursements was achieved from the SII (diary number 110/522/2018). All research procedures were employed in accordance with the relevant guidelines and regulations. Only de-identified register-based data were utilized, and study participants were not contacted, and therefore, consent from the patients was not needed according to Finnish legislation.

## Results

### Sample overview

The study cohort consisted of 4622 (53.8%) men and 3970 (46.2%) women with a mean disease duration of under 8 years (Table 1). At baseline, women achieved the LDL-C treatment target < 2.5 mmol/L less often than men (50.8% vs 55.7%, respectively, p < 0.001) and were less often on any statin treatment (56.1% vs 60.1%, respectively, p < 0.001). During the follow-up, 697 (15.1%) men and 537 (13.5%) women died. Comparisons of a few key indicators between the study cohort and the excluded patients are presented in Supplementary Table S5.

**Table 1:** Baseline characteristics by trajectory group for men and women.

Characteristics	Men						Women						sex *p*
	All men	Increasing	Decreasing	High-stable	Moderate-stable	Group *p*	All women	Increasing	Decreasing	High-stable	Moderate-stable	Group *p*	
Total	100 (4622)	2.5 (114)	3.5 (160)	6.0 (277)	88.1 (4071)		100 (3970)	2.6 (105)	4.3 (169)	9.7 (384)	83.4 (3312)		
**Sociodemographic characteristics**													
Age (years)	65.5 ± 10.6	63.4 ± 10.3	63.7 ± 10.8	60.4 ± 11.1	66 ± 10.4	< 0.001	68.6 ± 11.8	67.0 ± 10.7	66.5 ± 12.8	65.1 ± 13.4	69.1 ± 11.5	< 0.001	< 0.001
**Age groups**						< 0.001						< 0.001	< 0.001
50 or younger	8.2 (381)	8.8 (10)	12.5 (20)	17.3 (48)	7.4 (303)		7.3 (288)	6.7 (7)	9.5 (16)	14.8 (57)	6.3 (208)		
51–60	20.7 (959)	28.9 (33)	23.8 (38)	30 (83)	19.8 (805)		16.4 (651)	17.1 (18)	23.7 (40)	21.4 (82)	15.4 (511)		
61–70	38.8 (1794)	38.6 (44)	35.6 (57)	35.4 (98)	39.2 (1595)		29.6 (1174)	39 (41)	26.6 (45)	28.4 (109)	29.6 (979)		
71–80	24.8 (1148)	18.4 (21)	22.5 (36)	15.2 (42)	25.8 (1049)		29.9 (1187)	26.7 (28)	24.9 (42)	20.6 (79)	31.3 (1038)		
81 or older	7.4 (340)	5.3 (6)	5.6 (9)	2.2 (6)	7.8 (319)		16.9 (670)	10.5 (11)	15.4 (26)	14.8 (57)	17.4 (576)		
Time since diabetes diagnosis (years)	7.9 ± 6.4	7.3 ± 5.3	6.4 ± 5.4	5.3 ± 4.7	8.1 ± 6.5	< 0.001	7.7 ± 6.3	6.7 ± 5.3	7.2 ± 6.1	6.0 ± 5.7	7.9 ± 6.4	< 0.001	0.183
**Metabolic factors**													
BMI (kg/m^2^)^a^	31.2 ± 5.9	31.8 ± 6.6	32.2 ± 5.3	31.6 ± 6.3	31.1 ± 5.9	0.319	32.2 ± 6.7	33.1 ± 7.3	32.1 ± 5.6	32.5 ± 6.4	32.2 ± 6.8	0.760	< 0.001
Obese (BMI ≥ 30 kg/m^2^)^a^	52.9 (1292)	61.8 (34)	58.9 (43)	57.6 (68)	52.2 (1147)	0.234	60.6 (1329)	64.0 (32)	63.5 (54)	62.7 (131)	60.1 (1112)	0.774	< 0.001
HbA1c level (mmol/L)^b^	49.1 ± 13.4	50.2 ± 15	48.9 ± 14.4	49.1 ± 14.3	49.1 ± 13.3	0.918	48.1 ± 12.7	46.5 ± 10.7	53.2 ± 19	46.8 ± 12.9	48.0 ± 12.3	< 0.001	< 0.001
HbA1c level (%)^b^	6.7 ± 1.2	6.8 ± 1.4	6.6 ± 1.3	6.6 ± 1.3	6.7 ± 1.2	0.918	6.6 ± 1.2	6.4 ± 1.0	7.0 ± 1.7	6.4 ± 1.2	6.6 ± 1.1	< 0.001	< 0.001
HbA1c < 53 mmol/L (< 7.0%)^b^	72.2 (3080)	71.3 (72)	75.4 (101)	73.3 (173)	72 (2734)	0.819	76.3 (2828)	78.4 (76)	63.9 (99)	81.4 (280)	76.3 (2373)	< 0.001	< 0.001
**Statin treatment**													
Any statin	60.1 (2780)	69.3 (79)	45.0 (72)	42.2 (117)	61.7 (2512)	< 0.001	56.1 (2226)	64.8 (68)	43.8 (74)	34.4 (132)	58.9 (1952)	< 0.001	< 0.001
**Diagnosed comorbidity**													
Hypertension	28.9 (1335)	27.2 (31)	40.6 (65)	21.7 (60)	29.0 (1179)	< 0.001	33.2 (1319)	38.1 (40)	38.5 (65)	31.0 (119)	33.1 (1095)	0.249	< 0.001
Dyslipidemia	7.2 (331)	12.3 (14)	10.0 (16)	8.3 (23)	6.8 (278)	0.054	8.0 (318)	12.4 (13)	8.3 (14)	6.8 (26)	8.0 (265)	0.316	0.138
Ischemic CVDs	15.6 (720)	11.4 (13)	9.4 (15)	6.9 (19)	16.5 (673)	< 0.001	9.3 (370)	9.5 (10)	10.1 (17)	5.5 (21)	9.7 (322)	0.058	< 0.001
Atrial fibrillation	7.9 (363)	9.6 (11)	6.9 (11)	5.4 (15)	8.0 (326)	0.372	7.4 (292)	6.7 (7)	5.3 (9)	3.6 (14)	7.9 (262)	0.016	0.385
Heart failure	2.0 (92)	3.5 (4)	1.9 (3)	1.8 (5)	2.0 (80)	0.701	2.9 (115)	4.8 (5)	2.4 (4)	1.3 (5)	3.0 (101)	0.157	0.006
Peripheral arterial diseases	4.9 (226)	1.8 (2)	3.1 (5)	1.1 (3)	5.3 (216)	0.003	2.5 (101)	1.9 (2)	1.8 (3)	1.8 (7)	2.7 (89)	0.647	< 0.001
Chronic kidney diseases	3.0 (138)	5.3 (6)	3.1 (5)	1.4 (4)	3.0 (123)	0.227	1.9 (75)	1.0 (1)	0.6 (1)	0.5 (2)	2.1 (71)	0.070	0.001
Neuropathies	3.5 (162)	4.4 (5)	4.4 (7)	2.9 (8)	3.5 (142)	0.817	3.1 (122)	3.8 (4)	1.8 (3)	2.3 (9)	3.2 (106)	0.570	0.264
Retinopathy	2.8 (129)	1.8 (2)	3.1 (5)	2.5 (7)	2.8 (115)	0.895	1.8 (73)	1.9 (2)	3.0 (5)	1.8 (7)	1.8 (59)	0.744	0.004
Thyroid diseases	1.2 (54)	1.8 (2)	0 (0)	0.4 (1)	1.3 (51)	0.257	4.0 (150)	4.8 (5)	4.7 (8)	4.9 (19)	3.9 (128)	0.698	< 0.001
Concordant diseases and E11 subgroup diagnoses	48.9 (2261)	47.4 (54)	51.3 (82)	33.6 (93)	49.9 (2032)	< 0.001	46.8 (1857)	53.3 (56)	51.5 (87)	40.4 (155)	47.1 (1559)	0.021	0.047

Values are expressed as % (n) for categorical variables or mean ± SD for continuous variables.

*p* value comparisons across trajectory groups and sex are based on χ^2^ test for categorical variables and Kruskal–Wallis test for continuous variables.

^a^Number of missing BMI values for men (total: 2180, increasing: 59, decreasing: 87, high-stable: 159, moderate-stable: 1875) and women (total: 1776, increasing: 55, decreasing: 84, high-stable: 175, moderate-stable: 1462).

^b^Number of missing HbA1c values for men (total: 354, increasing: 13, decreasing: 26, high-stable: 41, moderate-stable: 274) and women (total: 262, increasing: 8, decreasing: 14, high-stable: 40, moderate-stable: 200).

### LDL-C trajectory groups

**Figure 1:**
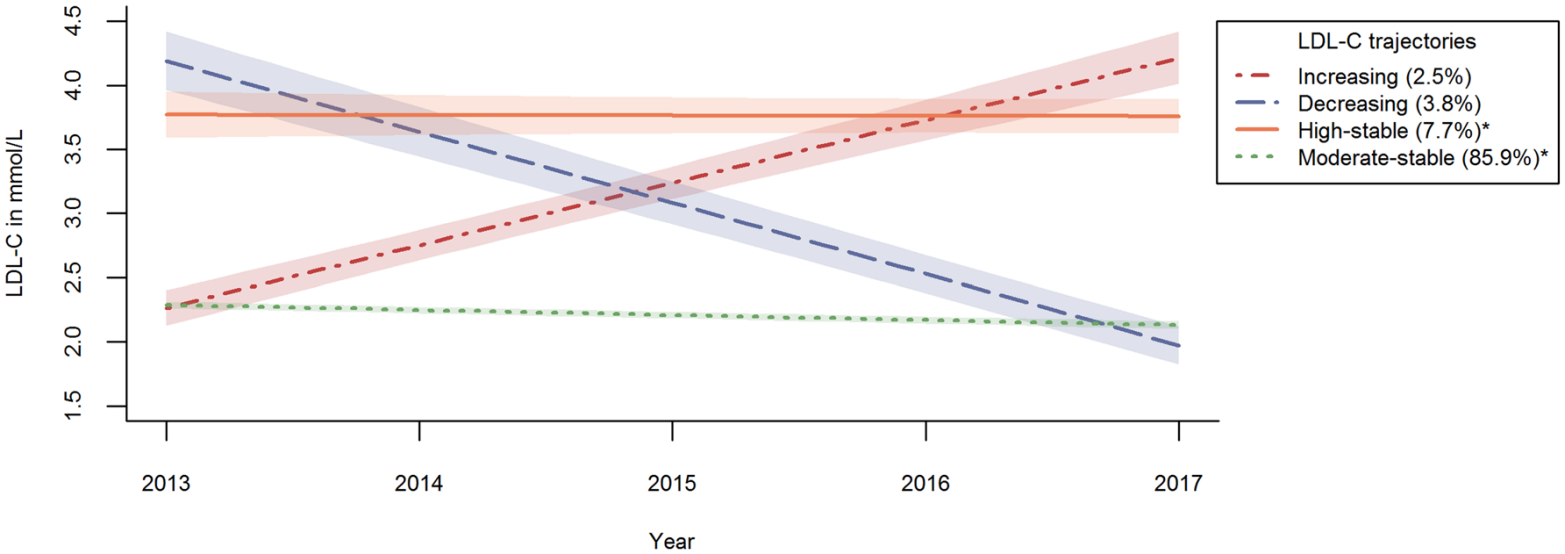
Four LDL-C trajectories, fitted with GMM. Proportion of men and women belonging to the LDL-C trajectories: **“**Increasing”: 2.5% men, 2.6% women (p = 0.601); “Decreasing”: 3.5% men, 4.3% women (p = 0.005), “High-stable”: 6.0% men, 9.7% women (p < 0.001), “Moderate-stable”: 88.1% men, 83.5% women (p < 0.001). *Statistically significant sex difference with p < 0.001.

A 4-group linear GMM with a random intercept factor was chosen as a final model over the 5-year follow-up time (Supplementary Table S6). The four identified LDL-C trajectory groups (Fig. 1) were named as “moderate-stable” (85.9%), “high-stable” (7.7%), “decreasing” (3.8%), and “increasing” (2.5%). Patients in the “moderate-stable” group had very stable LDL-C levels with a mean 2.2–2.3 mmol/L over the 2011–2017 follow-up period (Table 2). Stable LDL-C values also characterised the “high-stable” group although they were constantly elevated LDL-C levels (mean LDL-C over 3.9 mmol/L during 2011–2017) (Table 2). This group had the lowest annual and biennial LDL-C measurement rates and the lowest measurement frequency over the 2013–2017 follow-up period (Table 2) and was younger, more recently diagnosed with T2D and had less concordant comorbidities than the other groups (Table 1). The “decreasing” group was characterised by the highest measurement frequency (2013–2017) and initially high LDL-C levels at baseline (2011–2012) with a precipitous decline until the end of follow-up (2016–2017) from 3.3 to 1.9 mmol/L for men and from 3.5 to 2.1 mmol/L for women, respectively (Table 2). Hypertension and some other CVDs were more prevalent among patients belonging to this group. Patients in the “increasing” group showed an increase in LDL-C from 2.7 to 4.3 mmol/L in men and from 3.0 to 4.4 mmol/L in women between 2011–2012 and 2016–2017, respectively.

**Table 2:** LDL-C process and outcome indicators by trajectory group for men and women.

Indicators	Men						Women						Sex *p*
	All men	Increasing	Decreasing	High-stable	Moderate-stable	Group *p*	All women	Increasing	Decreasing	High-stable	Moderate-stable	Group *p*	
Total	100 (4622)	100 (114)	100 (160)	100 (277)	100 (4071)		100 (3970)	100 (105)	100 (169)	100 (384)	100 (3312)		
Mean follow-up time in years^a^	4.7 ± 0.9	4.9 ± 0.5	4.9 ± 0.6	4.8 ± 0.6	4.6 ± 0.9	0.002	4.7 ± 0.9	4.9 ± 0.5	4.7 ± 0.8	4.8 ± 0.7	4.7 ± 0.9	< 0.001	0.165
**Process indicators**													
LDL-C measurement frequency	4.7 ± 2.3	5.6 ± 2.8	6 ± 2.6	4.3 ± 2.4	4.6 ± 2.3	< 0.001	4.6 ± 2.3	5.4 ± 2.1	5.7 ± 2.9	4.4 ± 2.6	4.5 ± 2.2	< 0.001	0.082
Annual measurement	37.8 (1745)	35.1 (40)	46.3 (74)	22.7 (63)	38.5 (1568)	< 0.001	37.2 (1475)	39 (41)	42 (71)	29.4 (113)	37.7 (1250)	0.007	0.566
Biennial measurements	79.2 (3660)	80.7 (92)	86.3 (138)	69.3 (192)	79.5 (3238)	< 0.001	78.1 (3099)	81.9 (86)	85.8 (145)	68 (261)	78.7 (2607)	< 0.001	0.204
**Outcome indicators (mmol/L)**													
Overall LDL-C in 2011–2017^b^	2.39 ± 0.72	3.13 ± 0.64	3.14 ± 0.67	3.95 ± 0.52	2.23 ± 0.56	< 0.001	2.58 ± 0.79	3.45 ± 0.67	3.47 ± 0.82	4.01 ± 0.53	2.34 ± 0.55	< 0.001	< 0.001
< 2.5 mmol/L	60.5 (2797)	13.2 (15)	15.6 (25)	0 (0)	67.7 (2757)	< 0.001	51.3 (2036)	4.8 (5)	10.7 (18)	0 (0)	60.8 (2013)	< 0.001	< 0.001
< 1.8 mmol/L	20.1 (927)	0 (0)	0.6 (1)	0 (0)	22.7 (926)	< 0.001	13.4 (533)	0 (0)	0 (0)	0 (0)	16.1 (533)	< 0.001	< 0.001
LDL-C in 2011–2012 (baseline)^c^	2.44 ± 0.8	2.68 ± 0.89	3.32 ± 0.91	3.47 ± 0.9	2.34 ± 0.72	< 0.001	2.6 ± 0.87	3.01 ± 1.01	3.54 ± 1.1	3.58 ± 0.94	2.43 ± 0.73	< 0.001	< 0.001
< 2.5 mmol/L	55.7 (2360)	44.8 (47)	16.9 (23)	11.7 (28)	60.2 (2262)	< 0.001	50.8 (1863)	34.1 (31)	16.2 (24)	11.3 (40)	57.5 (1768)	< 0.001	< 0.001
< 1.8 mmol/L	19 (803)	14.3 (15)	4.4 (6)	2.1 (5)	20.7 (777)	< 0.001	14.3 (525)	7.7 (7)	3.4 (5)	2.3 (8)	16.4 (505)	< 0.001	< 0.001
LDL-C in 2013–2015^d^	2.32 ± 0.83	2.91 ± 0.97	3.14 ± 1.15	3.94 ± 0.71	2.17 ± 0.65	< 0.001	2.53 ± 0.91	3.28 ± 1.07	3.37 ± 1.27	4.03 ± 0.8	2.29 ± 0.66	< 0.001	< 0.001
< 2.5 mmol/L	63.3 (2836)	38.7 (43)	30.2 (48)	1.5 (4)	69.4 (2741)	< 0.001	54.7 (2100)	28.2 (29)	24.9 (42)	1.7 (6)	63.1 (2023)	< 0.001	< 0.001
< 1.8 mmol/L	25.3 (1133)	11.7 (13)	10.7 (17)	0 (0)	27.9 (1103)	< 0.001	18 (689)	1.9 (2)	11.2 (19)	0.3 (1)	20.8 (667)	< 0.001	< 0.001
LDL-C in 2016–2017^e^	2.27 ± 0.84	4.28 ± 0.74	1.98 ± 0.66	3.88 ± 0.61	2.1 ± 0.64	< 0.001	2.44 ± 0.89	4.39 ± 0.89	2.12 ± 0.68	3.95 ± 0.68	2.21 ± 0.62	< 0.001	< 0.001
< 2.5 mmol/L	66 (2466)	0 (0)	76.4 (110)	0 (0)	72.4 (2356)	< 0.001	58.4 (1867)	0 (0)	68.6 (96)	1.6 (5)	66.7 (1766)	< 0.001	< 0.001
< 1.8 mmol/L	29.6 (1104)	0 (0)	41 (59)	0 (0)	32.1 (1045)	< 0.001	21.9 (701)	0 (0)	32.9 (46)	0 (0)	24.7 (655)	< 0.001	< 0.001

Values are expressed as % (n) for categorical variables or mean ± SD for continuous variables.

Process indicators based on values during 2013–2017. Outcome indicators based on values during 2011–2017.

p value comparisons across trajectory groups and sex are based on χ2 test for categorical variables and Kruskal-Wallis test for continuous variables.

^a^Mean follow-up time between 1 Jan 2013 and end of follow-up (death, moving out of the region permanently or 31 Dec 2017, whichever came first).

^b^Mean of all valid measurements (2011–2017) starting from the baseline measurement if available.

^c^Number of patients with measurements in 2011–2012 for men (total: 4235, increasing: 105, decreasing: 136, high-stable: 239, moderate-stable: 3755) and women (total: 3667, increasing: 91, decreasing: 148, high-stable: 354, moderate-stable: 3074).

^d^Number of patients with measurements in 2013–2015 for men (total: 4479, increasing: 111, decreasing: 159, high-stable: 260, moderate-stable: 3949) and women (total: 3838, increasing: 103, decreasing: 169, high-stable: 360, moderate-stable: 3206).

^e^Number of patients with measurements in 2016–2017 for men (total: 3735, increasing: 106, decreasing: 144, high-stable: 230, moderate-stable: 3255) and women (total: 3196, increasing: 98, decreasing: 140, high-stable: 310, moderate-stable: 2648).

### Annual intensity of statin treatment

**Figure 2:**
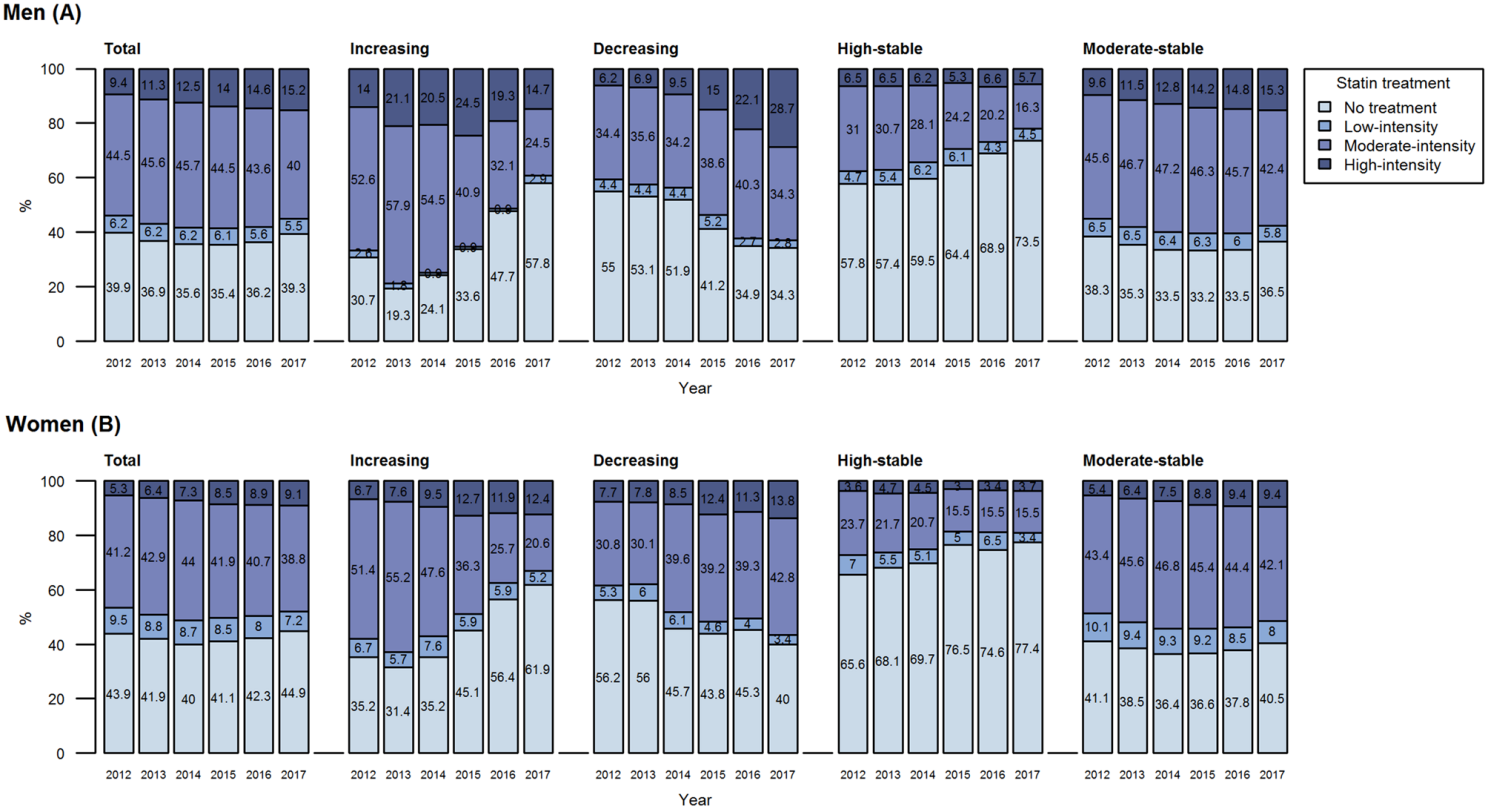
Annual statin treatment (%) by trajectory group for men (**A**) and women (**B**) (2012–2017). Each year’s plot only includes patients who were alive and living in North Karelia by 31 Dec. The number of patients by trajectory group and year is shown for men and women in Supplementary Table S5.

The group-specific statin treatment patterns were similar for both sexes during the 6-year follow-up 2012–2017; however, women were overall and in all groups more often untreated and less frequently treated with high-intensity statins than men (Fig. 2).

In the “moderate-stable” LDL-C group, the proportion of patients having any statin prescribed was relatively stable, with over 66.8% of men and 63.6% of women on treatment, whilst the proportion of high-intensity treatment increased in both sexes during the follow-up (Fig. 2). The “high-stable” LDL-C group had the lowest proportions of patients on moderate- and high-intensity treatment as well as any statin treatment. During the follow-up, the proportion of patients receiving any statin treatment decreased between 2012 and 2017 among men (42.3% vs 26.5%, respectively) and women (34.4% vs 22.6%, respectively).

The group with “decreasing” LDL-C levels showed changes in statin prescribing over the follow-up period: between 2012 and 2017, the proportion of patients without statin treatment decreased while there was an increase in high-intensity treatment among men (6.2% vs 28.7%, respectively) and women (7.7% vs 13.8%, respectively) and in moderate-intensity treatment among women (30.8% vs 42.8%). Changes were also observed in the “increasing” group where the proportion of patients with statin treatment declined from over 64% to less than 43%.

### Changes in statin treatment

For 63.1% of men and 64.5% of women, the intensity of statin treatment remained unchanged during the follow-up (“always unchanged”), almost one quarter had treatment intensified at some point (“ever intensified”) and about one-fifth de-intensified at some point (“ever de-intensified”) (Table 3). Women experienced statistically significantly fewer intensifications compared with men.

**Table 3:** Change in statin therapy between baseline (2012) and last year of follow-up by trajectory group for men and women.

Changes	Men						Women						Sex *p*
	All men	Increasing	Decreasing	High-stable	Moderate-stable	Group *p*	All women	Increasing	Decreasing	High-stable	Moderate-stable	Group *p*	
Total^a^	100 (4563)	100 (114)	100 (160)	100 (277)	100 (4012)		100 (3936)	100 (105)	100 (167)	100 (382)	100 (3282)		
**Change (at any time)**													
Ever intensified	24.6 (1122)	29.8 (34)	56.9 (91)	26 (72)	23.1 (925)	< 0.001	22.7 (894)	41 (43)	49.7 (83)	24.1 (92)	20.6 (676)	< 0.001	0.043
Time (years) to 1st intensification	2 [1–4]	1 [1–3]	3 [2–4]	2 [1–4]	2 [1–4]	< 0.001	2 [1–4]	2 [1–5]	2 [2–4]	2.5 [1–4]	2 [1–4]	0.076	0.827
Ever de‐intensified	19.5 (888)	49.1 (56)	27.5 (44)	37.5 (104)	17 (684)	< 0.001	20.4 (801)	62.9 (66)	28.1 (47)	31.2 (119)	17.3 (569)	< 0.001	0.305
Time (years) to 1st de‐intensification	3 [2–5]	4 [3–4.5]	3 [1.5–5]	3 [2–4]	3 [2–5]	0.173	3 [2–4]	3 [2–4]	2 [1–4]	3 [2–4]	2 [1–4]	< 0.001	0.325
Experienced both	7.1 (324)	14.0 (16)	16.3 (26)	14.4 (40)	6.0 (242)	< 0.001	7.5 (296)	26.7 (28)	16.2 (27)	14.7 (56)	5.6 (185)	< 0.001	0.458
Always unchanged	63.1 (2877)	35.1 (40)	31.9 (51)	50.9 (141)	65.9 (2645)	< 0.001	64.5 (2537)	22.9 (24)	38.3 (64)	59.4 (227)	67.7 (2222)	< 0.001	0.179
**Comparison (2012 vs last year)**													
Initiation among non-users	31.7 (577)	37.1 (13)	62.5 (55)	19.4 (31)	31.1 (478)	< 0.001	26.3 (455)	37.8 (14)	54.3 (51)	12.4 (31)	26.6 (359)	< 0.001	< 0.001
Discontinuation among users	21 (575)	54.4 (43)	27.8 (20)	62.4 (73)	17.7 (439)	< 0.001	23.9 (528)	61.8 (42)	32.9 (24)	59.1 (78)	19.9 (384)	< 0.001	0.013
**Magnitude of change (2012 vs last year)**^**b**^						< 0.001						< 0.001	< 0.001
Intensified (+ 3 levels)	2.8 (130)	5.3 (6)	12.5 (20)	2.2 (6)	2.4 (98)		2 (80)	1.9 (2)	6.6 (11)	1.3 (5)	1.9 (62)		
Intensified (+ 2 levels)	8.5 (386)	5.3 (6)	20 (32)	8.3 (23)	8.1 (325)		8.2 (322)	8.6 (9)	22.2 (37)	4.7 (18)	7.9 (258)		
Intensified (+ 1 level)	6.9 (316)	5.3 (6)	13.8 (22)	1.4 (4)	7.1 (284)		5.5 (216)	5.7 (6)	6 (10)	5 (19)	5.5 (181)		
Overall unchanged	67.2 (3068)	44.7 (51)	39.4 (63)	59.6 (165)	69.5 (2789)		69.3 (2729)	38.1 (40)	49.7 (83)	67.8 (259)	71.5 (2347)		
De-intensified (− 1 level)	3.1 (143)	2.6 (3)	2.5 (4)	4.7 (13)	3.1 (123)		4.1 (160)	11.4 (12)	4.2 (7)	4.2 (16)	3.8 (125)		
De-intensified (− 2 levels)	9.6 (440)	28.9 (33)	10.6 (17)	19.9 (55)	8.3 (335)		9.9 (388)	33.3 (35)	8.4 (14)	14.9 (57)	8.6 (282)		
De-intensified (− 3 levels)	1.8 (80)	7.9 (9)	1.3 (2)	4 (11)	1.4 (58)		1 (41)	1 (1)	3 (5)	2.1 (8)	0.8 (27)		
**Treatment among "overall unchanged"**	(n = 3068)	(n = 51)	(n = 63)	(n = 165)	(n = 2789)	< 0.001	(n = 2729)	(n = 40)	(n = 83)	(n = 259)	(n = 2347)	< 0.001	< 0.001
No treatment^c^	40.5 (1243)	43.1 (22)	52.4 (33)	78.2 (129)	38 (1059)		46.7 (1275)	57.5 (23)	51.8 (43)	84.6 (219)	42.2 (990)		
Low intensity	5.1 (155)	2 (1)	6.3 (4)	3.6 (6)	5.2 (144)		7.2 (197)	0 (0)	3.6 (3)	1.9 (5)	8.1 (189)		
Moderate intensity	44.9 (1377)	45.1 (23)	33.3 (21)	15.2 (25)	46.9 (1308)		40.9 (1117)	32.5 (13)	38.6 (32)	12 (31)	44.4 (1041)		
High intensity	9.6 (293)	9.8 (5)	7.9 (5)	3 (5)	10 (278)		5.1 (140)	10 (4)	6 (5)	1.5 (4)	5.4 (127)		

Values are expressed as % (n) for categorical variables, mean ± SD or median [interquartile range] for continuous variables.

*p* value comparisons across trajectory groups and sex are based on χ^2^ test for categorical variables and Kruskal–Wallis test for continuous variables.

^a^Patients who died or moved outside of the study region during 2013 (n = 99) were excluded from this analysis so that a patients’ last year of follow-up varied between 2013 and 2017.

^b^Graded treatment change (intensified or de-intesified) of 3 levels (change between "no treatment" and "high-intensity"), 2 levels (change between "no treatment" and "moderate-intensity", or between "low-" and "high-intensity"), and 1 level (change between "no treatment" and "low-intensity", between "low-" and "moderate-intensity", or between "moderate-" and "high-intensity").

^c^Other lipid-lowering treatment was used by ≤ 1.2% (men: ≤ 1.1%, women: ≤ 1.6%) of the patients with “overall unchanged” no statin treatment.

Trajectory groups with stable LDL-C patterns had the highest proportion of patients with “overall unchanged” statin treatment intensity. However, the “moderate-stable” group patients mainly remained at a moderate-intensity (over 44% of unchanged) whilst in the “high-stable” groups, patients mainly remained untreated (over 78% of unchanged) (Table 3). The highest proportion of treatment intensifications and initiations were observed in the “decreasing” group in which statin therapy was initiated for over half of the patients who were not on treatment in 2012 (Table 3). On the other hand, patients in the “increasing” group had the highest proportion of treatment de-intensifications and discontinuations. Over half of these patients with initial statin use discontinued the therapy by the end of follow-up.

## Discussion

### Statement of principal findings

We identified four distinct LDL-C trajectories (“increasing”, “decreasing”, “high-stable” and “moderate-stable”) which differed in terms of treatment practices. Most patients had a “moderate-stable” LDL-C trajectory with sufficiently regular follow-up visits and constantly high proportions of patients with moderate- and high-intensity statin treatment. Among these patients, the LDL-C treatment targets < 2.5 mmol/L and < 1.8 mmol/L were achieved at the end of follow-up by over two-thirds and one quarter of the patients, respectively. A key finding of our study was the identification of the second-largest patient group with “high-stable” LDL-C levels which did not receive proper medication and was poorly followed despite the increased need for regular testing. As the proportion of patients without statin treatment even increased in this group to over 73% in 2017, less than 2% of the patients achieved LDL-C < 2.5 mmol/L in at the end of follow-up. A small group of patients (“decreasing”) with initially high LDL-C levels managed to drastically reduce LDL-C so that in the end, over 68% and 32% had LDL-C below 2.5 mmol/L and 1.8 mmol/L, respectively. These patients were more regularly followed and statin therapy was initiated and intensified more frequently than in other groups. We also identified a small group of patients (“increasing”) that showed, despite being regularly monitored, a drastic increase in LDL-C along with the highest rates of de-intensification or discontinuation of treatment.

Women had worse LDL-C control, were less often prescribed statin, or had it prescribed at a lower intensity, and exhibited treatment discontinuations more often than men. No sex disparities were observed regarding measurement rates and overall measurement frequency. Women belonged more often to the “high-stable” LDL-C trajectory and less often to the “moderate-stable” LDL-C trajectory than men.

### Findings in relation to other studies

#### Trajectories identified

Only a few studies have applied trajectory modelling to LDL-C measurements with large differences between studies. Tsai et al. identified, comparable to the two stable trajectories in our study, two relatively stable LDL-C trajectories (with LDL-C levels decreasing 3.4–3.1 mmol/L and 2.3–2.2 mmol/L, mean 2.8 years of follow-up) among chronic kidney disease patients with patients in the lower LDL-C trajectory being older and more often male^17^. Pencina et al.^16^ identified three non-HDL-cholesterol trajectories along age among individuals without CVD and diabetes at baseline (mean 32.6 years of follow-up). Duncan et al.^15^ found five LDL-C trajectories (“optimal”, “borderline” and three elevated but decreasing trajectories) along age among individuals without exclusion criteria based on health status (22–30 years of follow-up). Information on statin use was only available in Duncan et al. study, identifying higher statin use at the end of follow-up (45.9–90.9%) as the main reason for the observed decrease in LDL-C towards the end of follow-up in the “elevated” trajectories.

#### Differences in diabetes care

Care processes and outcomes varied between the trajectory groups regarding LDL-C measurement activity, LDL-C outcomes and statin treatment. During the time of follow up, the Finnish Current Care Guideline recommended, in accordance with international guidelines, regular assessment of LDL-C levels every 1–3 years and LDL-C treatment targets of < 2.5 mmol/L for T2D patients with a high CVD risk, and < 1.8 mmol/L or a 50% reduction from baseline for patients with a very high CVD risk due to additional CVD risk factors^23^.

There were significant differences in the proportions of untreated patients and statin treatment intensifications (mainly initiations). The elevated LDL-C levels in the “high-stable” group would require initiating statin treatment and closer LDL-C monitoring. The looser diabetes management among patients in this group might be partially due to their relatively lower CVD risk based on the younger age and lower disease burden. Most baseline comorbidities were less often diagnosed among patients with high-stable LDL-C than in the other groups. According to two Finnish and one German studies, statin treatment is more frequent among the oldest age groups of an elderly population^13^, among newly diagnosed diabetes patients with a history of CHD compared with those without CHD^30^, and among atherosclerotic CVD patients including patients with diabetes compared with diabetes patients without atherosclerotic CVD^31^. In contrast, the higher rates of hypertension and some other CVDs might partially explain the drastic improvements in the “decreasing” group as more patients aim for the lower LDL-C treatment target. There were statistically significant differences in baseline HbA1c among women (highest values in the “decreasing” group) but no differences among men.

The trajectory groups also differed regarding de-intensification rates (mainly discontinuations) among initial statin users during the 6-year follow-up. Particularly striking was the decrease in statin prescriptions among patients with “increasing” and “high-stable” LDL-C levels. Considering that over half of the Finnish statin users discontinue the treatment without consulting a physician^32^, it is comprehensible that we observed lower discontinuation rates compared with studies using drug reimbursement data^33^. Interestingly, patients in the “increasing” group had a relatively good measurement activity which was not the case in the “high-stable” group.

Previous studies have found substantial under-treatment and delay in treatment initiation among patients recommended being treated with statins^9,34,35^. Considering the high proportions of treatment discontinuations, especially in the “increasing” group, omitted treatment re-initiation among patients whose prescription was not renewed or explicitly stopped is problematic. In practical clinical work, a common problem is to distinguish otherwise common muscle pains that occur incidentally during statin therapy from statin-associated muscle symptoms. Guidelines recommend to re-initiate another statin or to lower the dose if possible^7,25^. More than 70% of patients who stopped due to side effects tolerated the statin when it was restarted^36^.

Although we have no information on adherence, we hypothesise that not all patients took the prescribed medication correctly. Adherence issues especially apply to the “increasing” and “high-stable” group where almost nobody achieved LDL-C treatment target < 2.5 mmol/L in 2016–2017 although over a third and about a fifth, respectively, had had moderate- and high-intensity treatment prescribed. Adherence issues in the “high-stable” group are plausible as poor adherence is associated with lower age, being female and absence of hypertension^37^. In two Finnish studies, only about half of statin initiators were considered adherent during the first year based on data from the Finnish Prescription Register^38,39^.

Overall, our study observed clinically significant improvements regarding LDL-C levels along with statin treatment intensifications, resulting in achievement rates for LDL-C targets < 2.5 mmol/L and < 1.8 mmol/L of 66% and 30% for men, respectively, and 58% and 22% for women, respectively, at the end of follow-up. This development is in line with the Finnish Current Care Guideline, recommending the initiation of drug treatment simultaneously with lifestyle changes for patients with LDL-C levels above the treatment target, and the intensification of treatment if the treatment target is still not achieved after the initiation and the drug is well tolerated^7^. Ezetimibe is recommended as a second-line therapy if statins are not tolerated or the desired LDL-C reduction is not achieved^7^. Strikingly, LDL-C reduction was also observed in patients without treatment intensification or even without statin treatment at all, indicating that also other factors, such as lifestyle modifications, very high age or comorbidities, influenced care outcomes. In 2020, the Finnish Current Care Guidelines published new recommendations^40^ in accordance with the ESC/EAS 2019 guidelines^6^, lowering the LDL-C treatment targets for patients at moderate, high, and very high CVD risk to < 2.6 mmol/L, < 1.8 mmol/L, and < 1.4 mmol/L, respectively. The majority of T2D patients is considered to have high to very high CVD risk except those aged < 40–50 years with no other CVD risk factors. Efforts to reduce LDL-C must continue even in the improving LDL-C trajectory groups. Coinciding with previous studies^41^, special attention should be paid to women who less often meet LDL-C treatment targets.

There are many possible explanations for the gender difference in LDL-C control observed in our study. Based on previous studies, it may be caused by differences in statin dosages, adherence to statins, pathophysiology, or pharmacodynamics and pharmacokinetics between women and men^42,43^. Studies on individuals with diabetes have also found, that thyroid diseases are more common among women than men and are associated with higher LDL-C^44,45^. In our study, the prevalence of thyroid disease was three times higher among women than men but there was no statistically significant difference between the trajectory groups.

### Possible explanations and implications for clinicians and policymakers

Sociodemographic background factors influence the patient’s statin-related choices^37^ but experienced or feared adverse effects, perceived futility, lack of knowledge about their efficacy, and lack of practical support in their use might influence statin use even more^32,46^. Clinician warnings about the small risk of rhabdomyolysis and negative information in the media affect patients’ expectation of harm^47^. Complaints about statin-associated muscle aches are relatively common in clinical practice while the incidence in treatment studies with standard statin doses has been similar in the statin and placebo groups^47,48^.

Media coverage directly reduces statin adherence and increases discontinuations and even CVD mortality^49,50^. News stories are, as opposed to the prevailing scientific narrative, predominantly negative in lay media^51^, focusing on the main narratives of side effects and “over-medicalisation” of healthy people^52^. A survey across Finland revealed that nearly every third discontinuation was induced by public discussions about adverse effects^32^. The study showed that the knowledge on the benefit of statins should improve although it remained unclear whether it was due to a lack of counselling or a lack of understanding of the information given. Respondents who never used statins cited most typically “futility” (72%) and “the physician never proposed” (28%) from the listed rationales for not initiating statin therapy. Among current and former statin users, 31‒58% knew the treatment aims (primary or secondary prevention of CVDs) and only approximately half were aware of cholesterol treatment goals, of which 49% and 72% could not name a numerical value for total cholesterol and LDL-C, respectively^32^.

Patients’ fear of side effects constitutes the major challenge to the re-initiation of statin therapy^53^. Physicians may need to more actively and clearly address patients’ perceptions of treatment goals and feelings of necessity in clinician-patient risk/benefit discussions through patient-centred approaches such as motivational interviewing^54,55^ and offer reputable sources as an alternative to mass media. Care outcomes could futher be supported by defining personal care plans and providing paper copies^56^ in addition to the MyKanta online service, where Finnish patients can nationwide view their health data and prescriptions^57^.

Better knowledge of patient attitudes and behaviours and insights in care provision are needed to improve care processes. The use of pharmacy claims data could bring further understanding on the interplay between professional- and patient-related factors.

### Strengths and weaknesses

To our knowledge, this is the first study linking LDL-C trajectories to quality of care process indicators and patterns of lipid-lowering medication among T2D patients. Trajectory modelling allows for a visual representation of patterns over time. It demonstrates how repeated measures over a substantial follow-up time can be more useful than a simple single point or average measures in identifying and drawing attention to patients with insufficient care. GMM is a suitable method to analyse real-life routine-care data with missing data and unequally spread measurements. EHR data is not prone to non-responsiveness or recall bias.

Our study also had some limitations. The exclusion of patients with fewer measurements (15.7%) might reduce the results’ generalizability (Supplementary Table S5). The data did not include individuals who used only private healthcare services. The data did not contain information on socioeconomic factors, diet or physical activity. As our study focused on service provision using drug prescription data. It remains unknown whether patients redeemed prescriptions or the extent to which the medication supply via a recorded prescription was present on or shortly before the annual index date. Data quality depends on care and recording practices. We suspect, for instance, that dyslipidemia is underdiagnosed in our cohort as unexpected low prevalence among patients with critically high LDL-C suggests. Using routine care data for research is an important step to improve care and record processes, and ultimately improve the data quality^58^.

## Conclusion

Besides overall improvements in T2D management, a significant variation between LDL-C trajectories regarding LDL-C development as well as measurement activity and statin treatment were identified. The identified trajectory groups were associated with the existence and non-existence of statin treatment as well as treatment intensification and discontinuation. In view of the recent adaptation of lipid management guidelines, physicians should increase efforts to achieve the LDL-C treatment targets—especially in the patient group with constantly elevated LDL-C levels—by paying attention to earlier initiation of statin treatment, intensification of treatments when necessary and re-initiating if possible. The results of our study may support physicians to identify patients who need to be monitored more closely beyond a single time point measurement.

## Supplementary Information

Supplementary Information.

## Data Availability

The health records data analysed in the current study is confidential and, according to the Personal Data act, cannot be made publicly available to protect the privacy of the patients.

## Abbreviations

ATC: Anatomical therapeutic chemical
CVD: Cardiovascular diseases
EHR: Electronic health record
GMM: Growth mixture model
LCGA: Latent class growth model
LDL-C: LDL-cholesterol
SII: Social Insurance Institution
Siun Sote: Joint Municipal Authority for North Karelia Social and Health Services
T2D: Type 2 diabetes
