# Supplementary material for: LDL-cholesterol trajectories and statin treatment in Finnish type 2 diabetes patients: a growth mixture model
Source: Sci Rep. 2021 Nov 19;11:22603. doi: 10.1038/s41598-021-02077-6 (PMC8604948; doi:10.1038/s41598-021-02077-6)
Supplement: Supplementary file 1 — Supplementary Information. [file 41598_2021_2077_MOESM1_ESM.docx]

**Supplementary Information**

**Content
Tables**

[Supplementary Table S1 Number of patients with annual LDL-C measurement available 1](#_Toc70585022)

[Supplementary Table S2 Diagnosed concordant comorbidities 2](#_Toc70585023)

[Supplementary Table S3 Statin therapy intensity levels 2](#_Toc70585024)

[Supplementary Table S4 Number of patients included in each year’s plot by trajectory group for men and women 3](#_Toc70585025)

[Supplementary Table S5 Comparison between the study cohort (n = 8592) and type 2 diabetes patients excluded due to insufficient number of LDL-C measurements between baseline or 1 Jan 2013 and 31 Dec 2017 (n = 1547) 4](#_Toc70585026)

[Supplementary Table S6 Unadjusted continuous variables GMM for LDL-C values measured annually in 2013–2017 among T2D patients (n = 8592). 5](#_Toc70585027)

**Figures**

[Supplementary Figure S1 Flow chart of the final study cohort 7](#_Toc70585028)

Supplementary Table S1 Number of patients with annual LDL-C measurement available

| **Used for** | **Definition of measurement** | **Periods** | **Number of patients with measurements** |
| --- | --- | --- | --- |
| Trajectory modelling | Last measurement of each period | 2013 | 6642 |
|  |  | 2014 | 6369 |
|  |  | 2015 | 5998 |
|  |  | 2016 | 5762 |
|  |  | 2017 | 5421 |
| Outcome indicator | Last measurement of each period | 2011–2012 (baseline) | 7902 |
|  |  | 2013–2015 | 8317 |
|  |  | 2016–2017 | 6931 |
|  | Mean of all values, starting from baseline values (2011–2012) | 2011–2017 | 8592 |

Note: 1234 patients died, and 120 moved outside of the study region at some point during the follow-up between 1 Jan 2013 and 31 Dec 2017.

Supplementary Table S2 Diagnosed concordant comorbidities

| **Concordant disease** | **Definition (ICD-10 code diagnosed before 1 Jan 2013)** |
| --- | --- |
| Hypertension | I10 |
| Dyslipidemia | E78 |
| Ischemic cardiovascular diseases | 120-I25; I63-I66 (except I63.6) |
| Atrial fibrillation | I48 |
| Heart failure | I50, I11.0, I13.0, I13.2 |
| Peripheral arterial diseases | I70.2, I73.9, I179.2, E11.5 |
| Chronic kidney diseases | N18, N19, N08.3, E11.2 |
| Neuropathies | G59, G63, G73, G99, E11.4 |
| Retinopathy | H54, E11.3, H36, H28.0, H43.1, H45.0, H40.5, H42.0 |
| Thyroid diseases | E00-E07 |
| Concordant diseases and  E11 subgroup diagnoses | Any of these concordant diseases or E11 subgroup diagnoses E11.6-E11.8 |

Supplementary Table S3 Statin therapy intensity levels

| Intensity | Average LDL–C reduction | Classification |
| --- | --- | --- |
| No statin treatment | - | No available prescription |
| Low-intensity | <30% reduction | Fluvastatin 20–40 mg  Lovastatin 20 mg  Pravastatin 10–20 mg  Simvastatin 5–10 mg |
| Moderate-intensity | 30% to <50% reduction | Atorvastatin 10–20 mg  Fluvastatin 80 mg  Lovastatin 40 mg  Pravastatin 40 mg  Rosuvastatin 5–10 mg  Simvastatin 20–60 mg |
| High-intensity | ≥50% reduction | Atorvastatin 40–80 mg  Rosuvastatin 20–40 mg  Simvastatin 80 mg  Simvastatin 10–80 mg + Ezetimibe 10 mg |

Supplementary Table S4 Number of patients included in each year’s plot by trajectory group for men and women

|  | **Men** | **Women** |
| --- | --- | --- |
| **Total** |  |  |
| **2012** | 4622 | 3970 |
| **2013** | 4561 | 3934 |
| **2014** | 4438 | 3844 |
| **2015** | 4189 | 3633 |
| **2016** | 4064 | 3547 |
| **2017** | 3872 | 3366 |
| **Increasing** |  |  |
| **2012** | 114 | 105 |
| **2013** | 114 | 105 |
| **2014** | 112 | 105 |
| **2015** | 110 | 102 |
| **2016** | 109 | 101 |
| **2017** | 102 | 97 |
| **Decreasing** |  |  |
| **2012** | 160 | 169 |
| **2013** | 160 | 166 |
| **2014** | 158 | 164 |
| **2015** | 153 | 153 |
| **2016** | 149 | 150 |
| **2017** | 143 | 145 |
| **Increasing** |  |  |
| **2012** | 277 | 384 |
| **2013** | 277 | 382 |
| **2014** | 274 | 376 |
| **2015** | 264 | 361 |
| **2016** | 257 | 355 |
| **2017** | 245 | 349 |
| **Moderate-stable** |  |  |
| **2012** | 4071 | 3312 |
| **2013** | 4010 | 3281 |
| **2014** | 3894 | 3199 |
| **2015** | 3662 | 3017 |
| **2016** | 3549 | 2941 |
| **2017** | 3382 | 2775 |

Supplementary Table S5 Comparison between the study cohort (n = 8592) and type 2 diabetes patients excluded due to insufficient number of LDL-C measurements between baseline or 1 Jan 2013 and 31 Dec 2017 (n = 1547)

|  | **Men** | | | **Women** | | |
| --- | --- | --- | --- | --- | --- | --- |
|  | **Study**  **cohort** | **Excluded patients** | **p-value** | **Study cohort** | **Excluded patients** | **p-value** |
| **Total** | **4622** | **740** |  | **3970** | **807** |  |
| **Socio-demographic characteristics** |  |  |  |  |  |  |
| Age (years) at baseline | 65.5 ± 10.6 | 68.8 ± 14.1 | <0.001 | 68.6 ± 11.8 | 77.4 ± 13.8 | <0.001 |
| Age groups |  |  | <0.001 |  |  | <0.001 |
| ≤50 years | 8.2 (381) | 10.5 (78) |  | 7.3 (288) | 4.3 (35) |  |
| 51–60 years | 20.7 (959) | 19.6 (145) |  | 16.4 (651) | 9.0 (73) |  |
| 61–70 years | 38.8 (1794) | 22.3 (165) |  | 29.6 (1174) | 10.7 (86) |  |
| 71–80 years | 24.8 (1148) | 21.5 (159) |  | 29.9 (1187) | 20.9 (169) |  |
| ≥81 years | 7.4 (340) | 26.1 (193) |  | 16.9 (670) | 55.0 (444) |  |
| **Time since diabetes diagnosis (years)** | 7.9 ± 6.4 | 8.1 ± 7.1 | 0.600 | 7.7 ± 6.3 | 9.1 ± 7.8 | <0.001 |
| **LDL-C over the follow-up period** |  |  |  |  |  |  |
| Mean | 2.4 ± 0.7 | 2.5 ± 0.9 | 0.071 | 2.6 ± 0.8 | 2.7 ± 1.0 | 0.131 |
| <2.5 mmol/L | 60.5 (2797) | 56.5 (256) | 0.097 | 51.3 (2036) | 47.0 (232) | 0.070 |
| <1.8 mmol/L | 20.1 (927) | 20.5 (93) | 0.810 | 13.4 (533) | 17.8 (88) | 0.008 |
| No LDL-C measurement available | 0 (0) | 38.8 (287) | <0.001 | 0.0 (0) | 38.8 (313) | <0.001 |
| **Statin treatment** |  |  |  |  |  |  |
| Any statin | 60.1 (2780) | 32.6 (241) | <0.001 | 56.1 (2226) | 24.4 (198) | <0.001 |
| **Diagnosed comorbidity** **at baseline** |  |  |  |  |  |  |
| Hypertension | 28.9 (1335) | 16.2 (120) | <0.001 | 33.2 (1319) | 21.3 (172) | <0.001 |
| Concordant diseases and  E11 subgroup diagnoses | 48.9 (2261) | 35.4 (262) | <0.001 | 46.8 (1857) | 41.4 (334) | 0.005 |
| **Died during follow-up** | 15.1 (697) | 56.6 (419) | <0.001 | 13.5 (537) | 63.2 (510) | <0.001 |

Supplementary Table S6 Unadjusted continuous variables GMM for LDL-C values measured annually in 2013–2017 among T2D patients (n = 8592).

| **Model** | | **Random factors** | **Log-likelihood** | **No. of para-meters** | **AIC** | **BIC** | **Entropy** | **VLMR-LRT P** | **Min APP** | **Classes** | | | | |
| --- | --- | --- | --- | --- | --- | --- | --- | --- | --- | --- | --- | --- | --- | --- |
|  |  |  |  |  |  |  |  |  |  | **1** | **2** | **3** | **4** | **5** |
| **Linear** | **1-class** | i | -32283.9 | 8 | 64583.8 | 64640.3 | NA | NA | 1 | 100.0 |  |  |  |  |
|  | **1-class** | I,s | -32116.3 | 10 | 64252.6 | 64323.2 | NA | NA | 1 | 100.0 |  |  |  |  |
|  | **2-class** | i | -31840.3 | 11 | 63702.6 | 63780.2 | 0.890 | <0.001 | 0.816 | 96.2 | 3.8 |  |  |  |
|  | **2-class** | i,s | -31677.8 | 13 | 63381.6 | 63473.4 | 0.858 | 0.0003 | 0.832 | 94.1 | 5.9 |  |  |  |
|  | **3-class** | i | -31368.8 | 14 | 62765.6 | 62864.5 | 0.817 | 0.0085 | 0.783 | 88.0 | 7.8 | 4.2 |  |  |
|  | **3-class** | i,s | -31360.9 | 16 | 62753.8 | 62866.7 | 0.816 | <0.001 | 0.785 | 87.1 | 9.2 | 3.7 |  |  |
|  | **4-class** | i | -31140.8 | 17 | 62315.6 | 62435.6 | 0.830 | <0.001 | 0.724 | 85.9 | 7.7 | 3.8 | 2.5 |  |
|  | **4-class** | i,s | -31135.1 | 19 | 62308.1 | 62442.3 | 0.828 | 0.0047 | 0.718 | 85.7 | 7.5 | 4.1 | 2.7 |  |
|  | **5-class** | i | -31051.8 | 20 | 62143.6 | 62284.7 | 0.842 | 0.2245 | 0.726 | 85.0 | 8.2 | 3.9 | 2.7 | 0.3 |
|  | **5-class** | i,s | -31029.3 | 22 | 62102.6 | 62257.9 | 0.839 | 0.2940 | 0.711 | 84.5 | 7.9 | 4.6 | 2.8 | 0.3 |
| **Quadratic** | **1-class** | i | -32283.9 | 9 | 64585.8 | 64649.3 | NA | NA | 1 | 100.0 |  |  |  |  |
|  | **1-class** | i,s | -32116.3 | 11 | 64254.6 | 64332.2 | NA | NA | 1 | 100.0 |  |  |  |  |
|  | **2-class** | i | -31803.5 | 13 | 63632.9 | 63724.7 | 0.790 | 0.0038 | 0.824 | 89.3 | 10.7 |  |  |  |
|  | **2-class** | i,s | -31626.3 | 15 | 63282.7 | 63388.5 | 0.859 | 0.6188 | 0.826 | 94.1 | 5.9 |  |  |  |
|  | **3-class** | i | -31334.7 | 17 | 62703.4 | 62823.4 | 0.840 | 0.1055 | 0.799 | 91.0 | 4.6 | 4.4 |  |  |
|  | **3-class** | i,s | -31207.9 | 19 | 62453.8 | 62588.0 | 0.887 | 0.0005 | 0.830 | 93.8 | 4.0 | 2.2 |  |  |
|  | **4-class** | i | -30962.4 | 21 | 61966.9 | 62115.1 | 0.841 | 0.0016 | 0.788 | 89.2 | 3.8 | 3.6 | 3.4 |  |
|  | **4-class** | i,s | -30877.7 | 23 | 61801.4 | 61963.7 | 0.838 | 0.0004 | 0.749 | 85.4 | 9.7 | 3.2 | 1.7 |  |
|  | **5-class** | i | -30689.7 | 25 | 61429.4 | 61605.9 | 0.819 | 0.1582 | 0.718 | 83.1 | 9.0 | 3.2 | 2.9 | 1.9 |
|  | **5-class** | i,s | -30604.4 | 27 | 61262.9 | 61453.4 | 0.832 | 0.0028 | 0.743 | 83.6 | 9.0 | 3.2 | 2.7 | 1.5 |
| **Cubic** | **1-class** | i | -32283.8 | 10 | 64587.7 | 64658.3 | NA | NA | 1 | 100.0 |  |  |  |  |
|  | **1-class** | i,s | -32116.0 | 12 | 64256.0 | 64340.7 | NA | NA | 1 | 100.0 |  |  |  |  |
|  | **2-class** | i | -31754.7 | 15 | 63539.5 | 63645.4 | 0.903 | <0.001 | 0.830 | 96.6 | 3.4 |  |  |  |
|  | **2-class** | i,s | -31620.0 | 17 | 63274.0 | 63394.0 | 0.855 | 0.1984 | 0.825 | 93.8 | 6.2 |  |  |  |
|  | **3-class** | i | -31257.8 | 20 | 62555.5 | 62696.7 | 0.839 | 0.0358 | 0.809 | 90.2 | 5.7 | 4.1 |  |  |
|  | **3-class** | i,s | -31181.7 | 22 | 62407.4 | 62562.7 | 0.876 | 0.0017 | 0.849 | 93.2 | 3.6 | 3.1 |  |  |
|  | **4-class** | i | -30919.8 | 25 | 61889.6 | 62066.1 | 0.823 | 0.0014 | 0.751 | 84.3 | 8.9 | 3.9 | 2.9 |  |
|  | **4-class** | i,s | -30783.7 | 27 | 61621.5 | 61812.0 | 0.859 | 0.0036 | 0.817 | 90.6 | 4.5 | 2.8 | 2.1 |  |
|  | **5-class** | i | -30609.5 | 30 | 61278.9 | 61490.7 | 0.821 | 0.0932 | 0.734 | 82.8 | 9.2 | 3.2 | 3.0 | 1.8 |
|  | **5-class** | i,s | -30442.0 | 32 | 60948.0 | 61173.9 | 0.827 | <0.001 | 0.748 | 82.9 | 9.2 | 3.7 | 2.5 | 1.7 |

Quadratic and cubic models were rejected as they were unable to create groups that were distinguishable regarding treatment outcomes.
Abbreviations: APP, average posterior probability; BIC, Bayesian information criteria; i, intercept; LMR-LRT, Lo-Mendell-Rubin likelihood ratio test; NA, not available; s, slope.

Supplementary Figure S1 Flow chart of the final study cohort

Patients with medical diagnosis of type 2 diabetes prior 1 Jan 2013 n = 10 204

1 547 patients excluded due to insufficient number of LDL-C measurements between baseline or 1 Jan 2013 and 31 Dec 2017:

• No measurements (n = 600)
• Only 1 measurement (n = 895)
• ≥2 measurements but in the same year (n = 52)

Included in the final study cohort n = 8 592

65 patients excluded due to:

• Correction of the diagnosis to type 1 diabetes (n = 58)
• Death before 1 Jan 2013 (n = 7)

Type 2 diabetes patients, residing in North Karelia by 31 Dec 2012 n = 10 139
